# Breaking Boundaries: A Case Report of Giant Hiatal Hernia Housing Pancreas, Stomach, Small Bowel, and Colon

**DOI:** 10.7759/cureus.90317

**Published:** 2025-08-17

**Authors:** Jonathan R Forrest, Urmimala Chaudhuri, Evans Konney, Drew J Triplett

**Affiliations:** 1 Internal Medicine, Boonshoft School of Medicine, Wright State University, Dayton, USA; 2 Gastroenterology, Boonshoft School of Medicine, Wright State University, Dayton, USA

**Keywords:** hiatal hernia, hiatal hernia repair, large hiatal hernia, paraesophageal hiatal hernia, type iv hiatal hernia

## Abstract

Hiatal hernia (HH) is defined as the protrusion of abdominal contents through the esophageal hiatus of the diaphragm into the mediastinum. They are classified into categories I-IV. Type I, known as the sliding hernia, accounts for up to 90% of HH cases and are typically managed medically. Types II-IV combined account for less than 15% of HH cases. Types II-IV HH often require surgical repair, especially when symptomatic, due to the risk of complications such as gastric volvulus, obstruction, or strangulation. Furthermore, pancreatic herniation in a type IV HH has been shown to cause acute pancreatitis. We present the case of a 84-year-old female patient with gastroesophageal reflux disease (GERD) and type IV HH who presented with a three-day history of chest pain, abdominal pain, nausea, and vomiting. Urgent abdominopelvic computed tomography (CT) revealed a large HH containing the entire stomach, portions of the duodenum, colon, small bowel loops, and almost the entire pancreas - a combination that is rarely seen. The patient’s symptoms resolved with ondansetron, fentanyl, and fluids, and she was discharged home from the ED with recommendations for gastroenterology follow-up. Although surgical management is considered definitive repair for type IV HH, this case highlights the possibility of non-operative medical management in chronic cases where the patient does not exhibit signs of ischemia, obstruction, volvulus, strangulation, or incarceration of the hernia. This case also highlights the importance of considering HH as a potential cause of chest pain, abdominal pain, nausea, and/or vomiting, especially in those with a history of GERD and already existing HH.

## Introduction

Hiatal hernia (HH) is defined as a protrusion of contents of the abdominal cavity through the esophageal hiatus of the diaphragm into the mediastinum [1]. Key risk factors for the development of HH include high body mass index (BMI) and advanced age, as well as congenital skeletal anomalies (related to bone degeneration and decalcification) or connective tissue disorders [2]. It has been shown that patients with a BMI >30 kg/m² have a four to five times higher risk of developing HH compared to those with normal BMI. HH are relatively common in the general population, with occurrence in approximately 55%-60% of individuals over age 50, although only 9% are symptomatic [1]. They are most common in women, as well as in North America and West Europe [1]. They are classified into categories I-IV [3]. Type I, known as the sliding hernia, accounts for up to 90% of HH cases and are typically managed medically. Types II-IV combined account for less than 15% of HH cases [3]. Types II-IV HH often require surgical repair, especially when symptomatic, due to the risk of complications involving the stomach and other involved abdominal organs [4]. Even more uncommon are giant or massive hernias, which some authors define as ones where >30% of the stomach herniates into the thoracic cavity and/or involves passage of other abdominal structures into the thorax [5]. Multiple modalities are utilized in the diagnosis of HH, including barium esophagram, computed tomography (CT), high-resolution esophageal manometry (HREM), and upper endoscopy. Barium esophagram has shown to yield a sensitivity of 76.8% for HH detection and often provides visualization of the herniated organs and region, as well as anatomic characterization of the gastroesophageal junction [6]. Contrast-enhanced CT is the most effective modality for diagnosis and characterization of HH due to short acquisition time, ease of use, and ability to directly identify the hiatal defects [7]. We present a rare case of a medically managed chronic type IV paraesophageal hernia involving the entire stomach and additional segments of the GI tract. The purpose of presenting this case is to highlight the possibility of non-operative medical management in chronic cases where the patient does not exhibit signs of ischemia, obstruction, volvulus, strangulation, or incarceration of the hernia.

This article was previously presented as a meeting abstract at the 2024 ACG Annual Meeting on October 27, 2024.

## Case presentation

**Figure 1:**
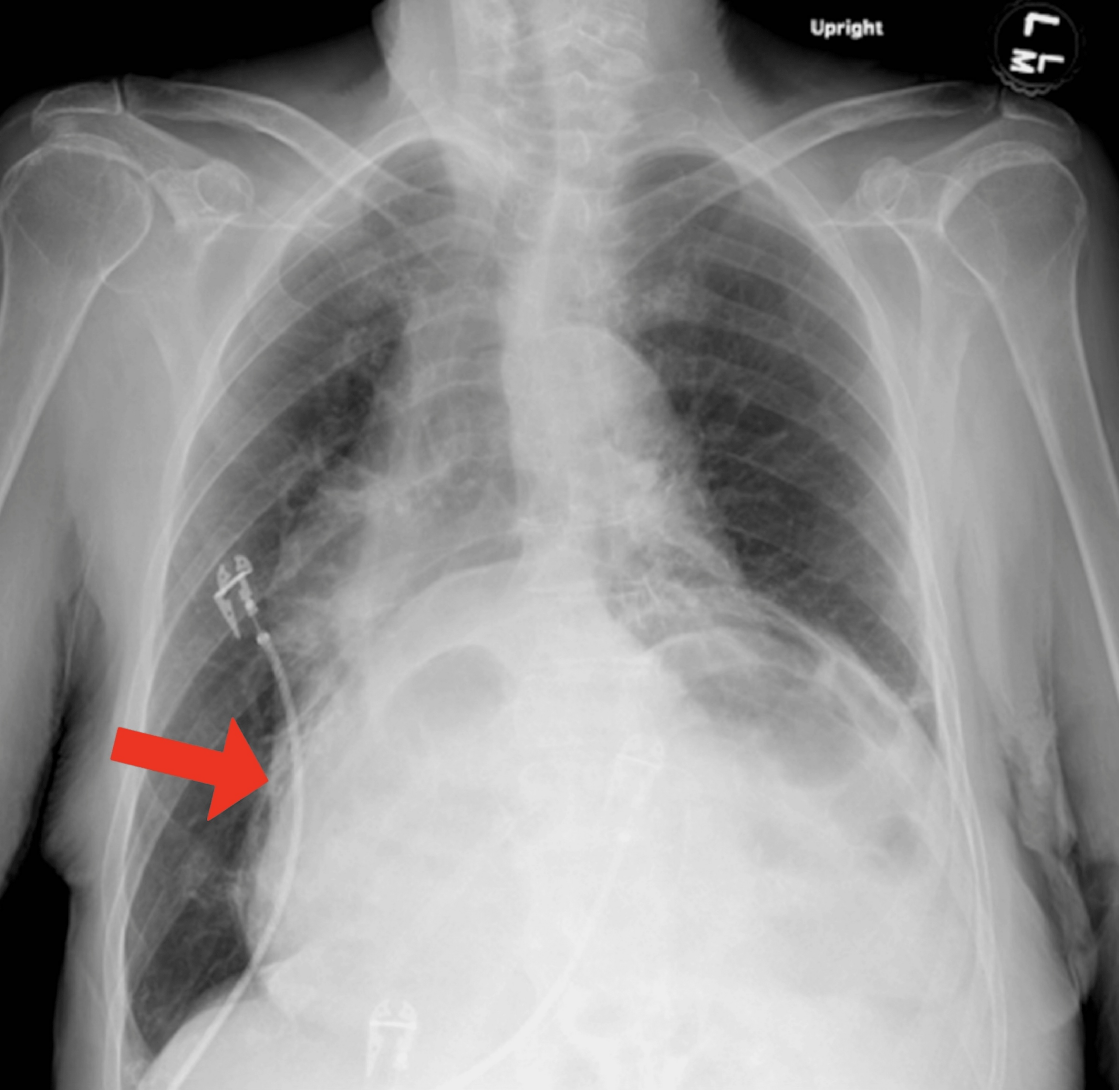
Chest X-ray showing large hiatal hernia sac (red arrow) with mediastinal deviation.

**Figure 2:**
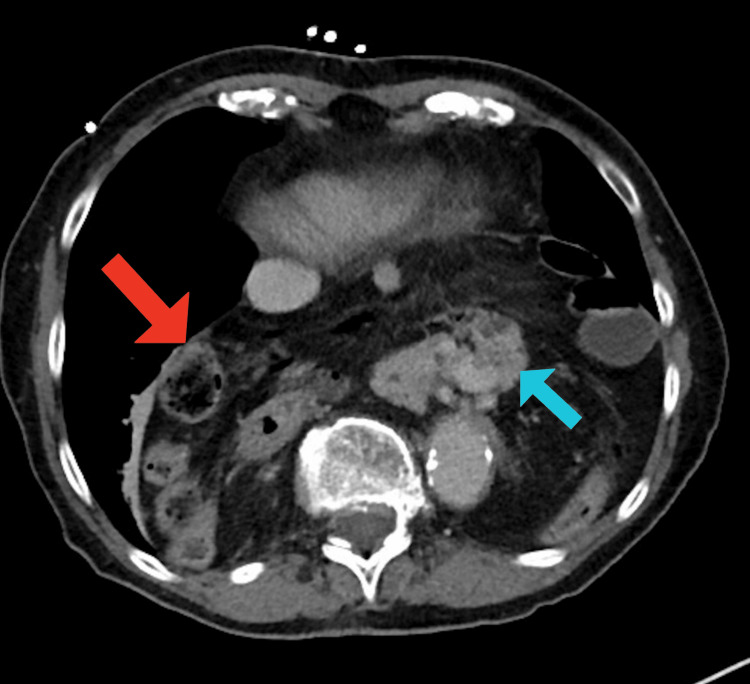
Axial CT abdomen and pelvis with contrast illustrating very large hiatal hernia sac (red arrow) containing pancreas (blue arrow), stomach, colon, and small bowel. CT: computed tomography

**Figure 3:**
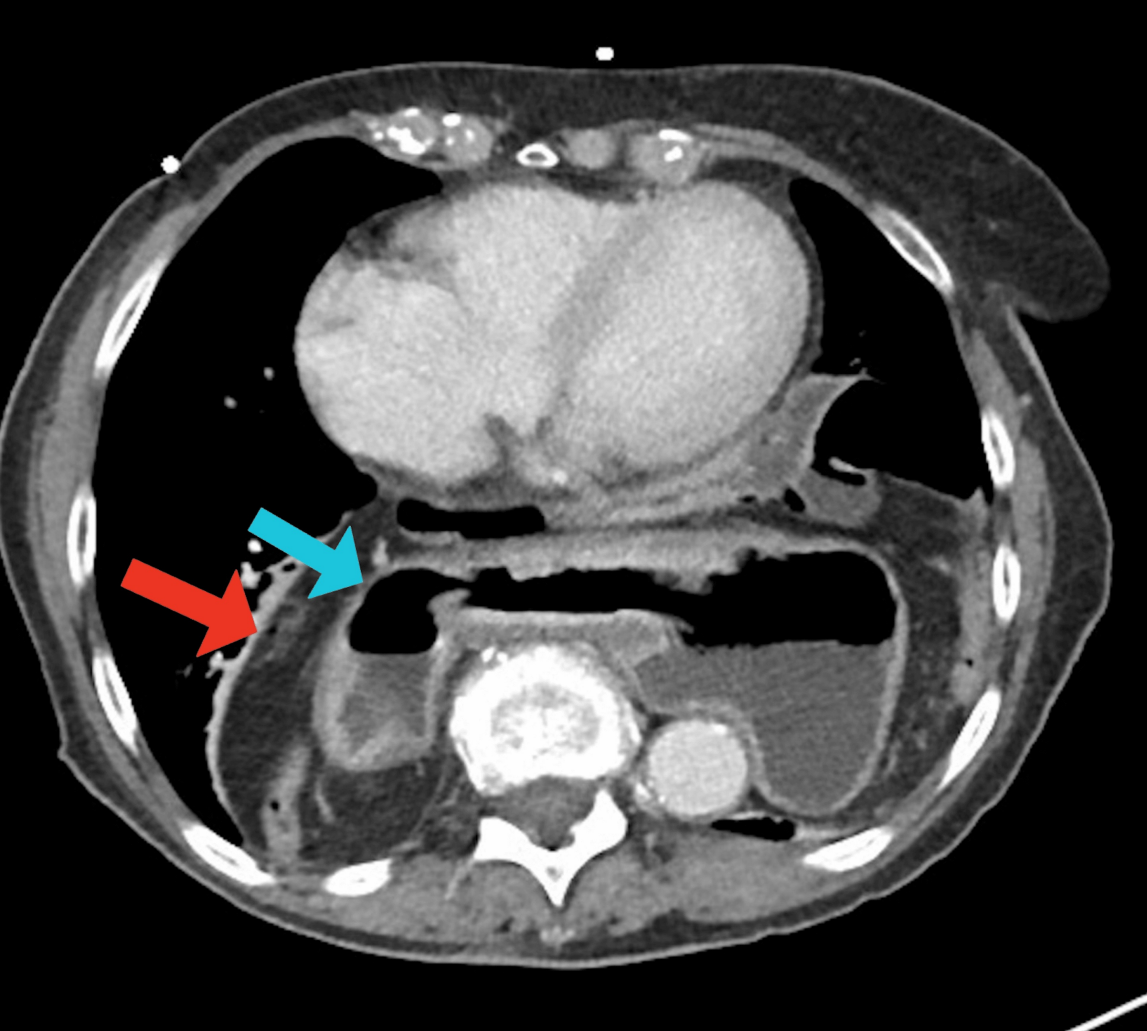
Axial CT abdomen and pelvis with contrast illustrating very large hiatal hernia sac (red arrow) containing stomach (blue arrow), colon, small bowel, and pancreas. CT: computed tomography

**Figure 4:**
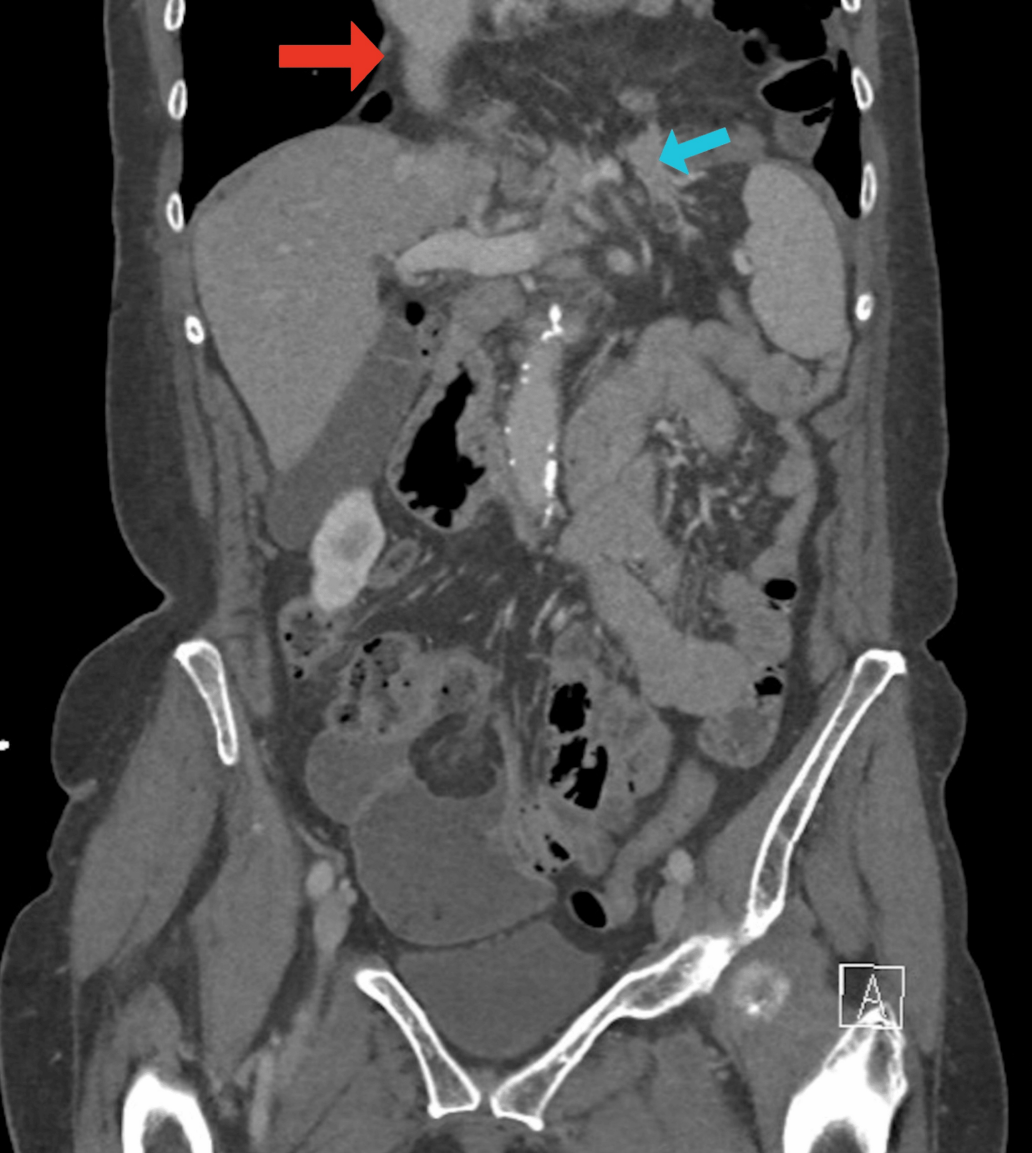
Coronal CT abdomen and pelvis with contrast image illustrating very large hiatal hernia sac (red arrow) containing pancreas (blue arrow), stomach, colon, and small bowel. There is a small volume of free fluid in the right pelvis that is non-specific. CT: computed tomography

An 84-year-old female patient with a past medical history of gastroesophageal reflux disease (GERD), type IV HH, and prior hysterectomy presented to the ED with chest pain, abdominal pain, nausea, and vomiting for three days. Initial vital signs were: temperature 98.1℉, blood pressure 156/82 mmHg, pulse 70 bpm, respiratory rate 16/min, and oxygen saturation 96% on room air. Abdominal examination showed a soft, non-distended abdomen with mild diffuse tenderness to palpation without rebound tenderness or guarding. Heart and lung examination gave unremarkable findings. Laboratory findings were remarkable for a hemoglobin of 16.2 g/dL (likely hemoconcentration due to vomiting) and glucose of 142 mg/dL. Other laboratory values, including troponin, liver function tests, and lipase, were within normal limits (Table 1). Chest X-ray showed a large HH with mediastinal deviation (Figure 1). Urgent abdominopelvic CT revealed a large HH containing the entire stomach, portions of the duodenum, colon, small bowel loops, and almost the entire pancreas (Figures 2-4). There were no signs of bowel ischemia or pneumoperitoneum. These findings were consistent with previous CT scans performed three years earlier; this lack of progression provided reassurance towards a stable, chronic condition. Additionally, she had never had a prior intervention for the HH, was always asymptomatic, and never had a prior medical encounter for the HH. The patient’s symptoms resolved with intravenous fluids, ondansetron, and fentanyl, and she was discharged home from the ED with ondansetron and recommendations for gastroenterology follow-up. Hospitalization was not indicated as the patient did not exhibit signs of ischemia, obstruction, volvulus, strangulation, or incarceration of the HH. Additionally, immediate operative intervention was deferred due to evidence showing the lack of post-surgical benefit at the patient’s age, as well as high risk for surgical complications at her age.

**Table 1:** Patient's laboratory values at initial presentation.

Analyte	Value	Reference ranges
Hemoglobin (g/dL)	16.2	Male: 13.5-17.5
		Female: 12.0-15.5
Glucose (mg/dL)	142	70-100
Troponin T (baseline) (ng/L)	14	≤14
Troponin T (1 hour) (ng/L)	13	≤14
Total bilirubin (mg/dL)	0.8	0.3-1.0
Aspartate aminotransferase (U/L)	18	10-40
Alanine aminotransferase (U/L)	12	10-40
Alkaline phosphatase (U/L)	69	30-120
Lipase (U/L)	54	13-60

## Discussion

HH are a common occurrence in the general population. They are graded from type I-IV. Type I (sliding) hiatal hernia occurs when the hiatal tunnel widens and the phrenoesophageal membrane loosens, causing part of the gastric cardia to move upward [3]. Type II is caused by a localized defect in the phrenoesophageal membrane, with the gastroesophageal junction staying fixed while the gastric fundus herniates [3]. Type III combines elements of both Type I and II, with the gastroesophageal junction moving above the diaphragm as the hernia enlarges. Type IV involves a large phrenoesophageal defect, allowing other organs like the colon, spleen, or pancreas to enter the hernia sac [3]. Pancreatic herniation in a type IV HH has been shown to cause acute pancreatitis. Among types I-IV, the most frequent HH is type I, which accounts for up to 90% of HH cases. Types II-IV combined account for less than 15% of HH cases [3]. Giant or massive hiatal hernias are even more rare; some authors define these as ones where >30% of the stomach herniates into the thoracic cavity and/or involves passage of other abdominal structures into the thorax [5]. These giant HH are estimated to represent 0.3%-15% of all HH [5]. Our patient met this criteria for giant HH. Multiple modalities are utilized in the diagnosis of HH, including barium esophagram, CT, HREM, and upper endoscopy. Barium esophagram was shown to yield a sensitivity of 76.8% for HH detection and often provides visualization of the herniated organs and region, as well as anatomic characterization of the gastroesophageal junction. [6,7]. CT is the most effective modality for diagnosis and characterization of HH due to short acquisition time, ease of use, and ability to directly identify the hiatal defects [7]. HREM is essential in pre-operative evaluation of HH repair to exclude motility disorders, such as achalasia [8]. The utility of upper endoscopy is limited, as it is often unable to appreciate larger HH [8].

The optimal management of type IV HH in poor surgical candidates is complex and subject to clinical judgment. For type II-IV HH, especially in symptomatic patients and those unresponsive to medical management, emergency or elective surgery is generally required to avoid life-threatening complications [7,9]. Some of these serious complications include volvulus (which can lead to bowel necrosis), obstruction, and even direct compression of the heart leading to compromised cardiac function [10]. It is also crucial to note that patients undergoing emergent HH surgery experience higher rates of major complications and a poorer prognosis compared to those undergoing elective repair [11]. Current guidelines suggest that surgical intervention should be decided on a case-by-case basis, taking into account patient comorbidities, age, and surgical risk [12]. The Stylopoulos study used a Markov Monte Carlo decision analysis model to simulate a hypothetical group of patients with asymptomatic or mildly symptomatic paraesophageal hernia, evaluating potential clinical outcomes under two treatment strategies: elective laparoscopic paraesophageal hernia repair versus watchful waiting [12]. The model suggests that surgical repair benefits 20% of patients under the age of 65; however, only 10% of patients over the age of 80 benefit from elective repair [12]. Therefore, it is reasonable to suggest that operative intervention should be pursued in patients up to 70-75 years of age regardless of symptomatic or asymptomatic nature. However, patients over the age of 75 should only undergo operative intervention if symptomatic, and should still be decided on a case-by-case basis [12]. If surgery is not pursued, recent data suggest that large HH patients managed conservatively have a low risk of hernia-related death and complications [13]. Thus, for asymptomatic patients, conservative therapy is an appropriate management strategy [13]. In terms of long-term surveillance for conservatively managed and asymptomatic HH, there is no currently accepted consensus; more research may be needed in this area. In our case, the patient did not have any symptoms including obstruction, volvulus, ischemia, or perforation, necessitating immediate surgical evaluation, and imaging comparison determined the hernia had not progressed (anatomically and complication-wise) for several years. Furthermore, due to minimal expected benefit and operative risk in our patient primarily due to age [12,13], medical management was ultimately deemed the preferred approach. In conclusion, surgical management is still recommended for definitive management of type IV HH, but our case represents a rare case of a chronically stable (defined as unchanged imaging findings) giant HH with periodic symptomatic medical management.

## Conclusions

To conclude, type IV HH is a rare condition that warrants prompt diagnosis and management. The purpose of presenting this case is to keep large HH on the differential in a patient presenting with non-specific symptoms, especially if they have a history of an already present type IV HH and/or GERD. Although surgical management is considered definitive repair for type IV HH to avoid life-threatening complications, this case highlights the importance of individualized, non-operative medical management in elderly patients who do not exhibit signs of ischemia, obstruction, volvulus, strangulation, or incarceration of the hernia.

## References

[1] Smith RE, Sharma S, Shahjehan RD (2024). Hiatal hernia. StatPearls.

[2] Yu HX, Han CS, Xue JR, Han ZF, Xin H (2018). Esophageal hiatal hernia: risk, diagnosis and management. Expert Rev Gastroenterol Hepatol.

[3] Kahrilas PJ, Kim HC, Pandolfino JE (2008). Approaches to the diagnosis and grading of hiatal hernia. Best Pract Res Clin Gastroenterol.

[4] Banimostafavi ES, Tayebi M (2018). Large hiatal hernia with pancreatic body herniation: case-report. Ann Med Surg (Lond).

[5] (006314). Giant hiatal hernia - The annals of thoracic surgery. https://www.annalsthoracicsurgery.org/article/S0003-4975(10)00631-4/fulltext.

[6] Weitzendorfer M, Köhler G, Antoniou SA (2017). Preoperative diagnosis of hiatal hernia: barium swallow X-ray, high-resolution manometry, or endoscopy?. Eur Surg.

[7] Nugraha HG, Agustina M, Nataprawira HM (2025). Diagnostic challenges of hiatal hernia Type IV: An imaging perspective. Radiol Case Rep.

[8] Sfara A, Dumitrascu DL (2019). The management of hiatal hernia: an update on diagnosis and treatment. Medicine Pharm Rep.

[9] Håkanson B, Lundell L, Rouvelas I, Thorell A (2018). The large hiatal hernia should be acknowledged and respected (in Swedish). Lakartidningen.

[10] Benelli ND, Altom B, Gomez-Garzon J, Matamoros J, Perez-Bello S, Castellanos L, Bello C (2024). Type IV paraesophageal hernia with posterior and inferior rotation of the stomach: a case report. Cureus.

[11] Baison GN, Aye RW (2020). Complex and acute paraesophageal hernias - type IV, strangulated, and irreducible. Annals of Laparoscopic and Endoscopic Surgery.

[12] Collet D, Luc G, Chiche L (2013). Management of large para-esophageal hiatal hernias. J Visc Surg.

[13] Oude Nijhuis RA, Hoek MV, Schuitenmaker JM, Schijven MP, Draaisma WA, Smout AJ, Bredenoord AJ (2020). The natural course of giant paraesophageal hernia and long-term outcomes following conservative management. United European Gastroenterol J.

